# Development of a synchronous recording and photo-stimulating electrode in multiple brain neurons

**DOI:** 10.3389/fnins.2023.1195095

**Published:** 2023-06-13

**Authors:** Qingping Zhang, Wei Jing, Shiping Wu, Mengzheng Zhu, Jingrui Jiang, Xiang Liu, Dian Yu, Long Cheng, Bin Feng, Jianbin Wen, Feng Xiong, Youming Lu, Huiyun Du

**Affiliations:** ^1^Department of Physiology, School of Basic Medicine, Tongji Medical College, Huazhong University of Science and Technology, Wuhan, China; ^2^Wuhan Center for Brain Science, Huazhong University of Science and Technology, Wuhan, China; ^3^Department of Pathophysiology, School of Basic Medicine, Huazhong University of Science and Technology, Wuhan, China; ^4^Department of Neurobiology, School of Basic Medicine, Tongji Medical College, Huazhong University of Science and Technology, Wuhan, China; ^5^Key Laboratory of Magnetic Resonance in Biological Systems, State Key Laboratory of Magnetic Resonance and Atomic and Molecular Physics, Wuhan Institute of Physics and Mathematics, National Center for Magnetic Resonance in Wuhan, Innovation Academy of Precision Measurement Science and Technology, Chinese Academy of Sciences, Wuhan, China; ^6^Hubei Key Laboratory of Drug Target Research and Pharmacodynamic Evaluation, Wuhan, China

**Keywords:** electrophysiological recording, optogenetic, multichannel recording, default mode network, brain network, neural circuit

## Abstract

The investigation of brain networks and neural circuits involves the crucial aspects of observing and modulating neurophysiological activity. Recently, opto-electrodes have emerged as an efficient tool for electrophysiological recording and optogenetic stimulation, which has greatly facilitated the analysis of neural coding. However, implantation and electrode weight control have posed significant challenges in achieving long-term and multi-regional brain recording and stimulation. To address this issue, we have developed a mold and custom-printed circuit board-based opto-electrode. We report successful opto-electrode placement and high-quality electrophysiological recordings from the default mode network (DMN) of the mouse brain. This novel opto-electrode facilitates synchronous recording and stimulation in multiple brain regions and holds promise for advancing future research on neural circuits and networks.

## Introduction

The mammalian brain is a highly complex yet intricately organized organ that consists of functionally interactive and structurally connected circuits and networks. These networks support various essential functions such as cognition, sensory perception, and motor control (Bassett and Bullmore, 2009; Bullmore and Sporns, 2012; Whitesell et al., 2021). Conversely, dysfunctions in these organized circuits/networks have been associated with psychiatric and neurological disorders (Buckner et al., 2008; Greicius, 2008). Advancements in functional magnetic resonance imaging (fMRI) have significantly contributed to our understanding of network/circuit organization in both healthy and diseased brains (Grandjean et al., 2020). However, fMRI’s limited temporal resolution and lack of behavioral synchronization often restrict its application in more detailed mechanistic studies (Nair et al., 2018; Jing et al., 2021). Invasive electrophysiological recordings and synchronized optogenetic stimulations are necessary for a deeper understanding of these complex systems, but ethical issues make them currently inaccessible for human studies. Therefore, researchers often opt for rodent models, especially genetically modified mice, to perform invasive electro- and opto-recording/stimulation for physiological and pathological studies of brain circuits/networks (Grandjean et al., 2020).

Flexible electrode grids and penetrating probes are commonly utilized to record electrocorticography (ECoG) and individual spikes/local field potentials (LFPs) in the brain, respectively (Gunasekera et al., 2015). Although flexible electrode grids offer benefits such as long-term recording and minimal brain tissue damage (Gunasekera et al., 2015; Ji et al., 2020; Liu et al., 2020), they are limited to the cortical surface and have restricted applicability in subcortical network/circuit research (Grandjean et al., 2020). Penetrating probes such as the Michigan array (Vetter et al., 2004), Utah array (Kim et al., 2006), Neuropixels probe (Jun et al., 2017; Juavinett et al., 2019; Steinmetz et al., 2021), and polymer electrode (Chung et al., 2019), allow for multi-site brain recordings simultaneously (Liu et al., 2020). However, these regularly shaped electrodes are not optimal for irregularly distributed brain areas within networks/circuits (e.g., default mode network, DMN). The mouse model, with established genetic manipulation approaches, is widely used in brain research, but implanting multiple probes sequentially in dispersed regions of the rodent brain remains challenging (Young and McNaughton, 2009; Jing et al., 2017, 2021; Cui et al., 2018, 2021; Lozano-Montes et al., 2020). Recent years have seen the development of multi-site drive systems targeting several brain regions (usually 3–8 areas), but their size and weight (usually >7 g) limit their application to rats (Headley et al., 2015; Billard et al., 2018; Ma et al., 2019; Sheng et al., 2021). Additionally, it is crucial to control cell-type-specific neural activities using optogenetics online and simultaneously monitor functional brain networks/circuits to uncover the mechanisms underlying healthy and diseased networks/circuits (Bernal-Casas et al., 2017; Grandjean et al., 2019; Lozano-Montes et al., 2020). Hence, there is a growing demand for compact opto-electrodes that integrate dispersed electrode wires and optical fibers.

In this study, we present a compact and ultra-light opto-electrode that can record and stimulate multiple widely distributed brain regions simultaneously. We aimed to develop a mass production program that reduces the workload of fabricating and implanting the opto-electrode. To achieve this, we employed a micro-nanofabrication quartz glass module to aid in laser-cutting electrode microwires, a customized laser-perforated steel sheet array to assemble the microwires, and a customized printed circuit board (PCB) to fix the microwire/optical fiber and connect the microwire to the connector. We tested the opto-electrode on freely moving mice to evaluate its implantation accuracy, recording stability, and responsiveness to light stimulation.

## Materials and methods

### Animals

For our electrode test, we selected male ChAT-Cre knock-in mutant mice (#006410, Jackson Laboratory, Bar Harbor ME, United States) and C57 mice that were 12 ± 1 weeks old. Breeding and rearing of these mice were conducted in accordance with the Animal Care and Use Committee’s protocols and institutional guidelines at Huazhong University of Science and Technology, Wuhan, China. The mice were group-housed with three to five per cage and subjected to a 12-h light–dark cycle, with the lights on at 8:00 am. The temperature and humidity of the mouse facility were maintained at a constant 21 ± 1°C and 50 ± 5%, respectively. Following electrode implantation, all mice were housed individually.

### The electrode fabrication

The fabrication of electrodes can be divided into the following process.

#### Preparation of microwires

**Figure 1:**
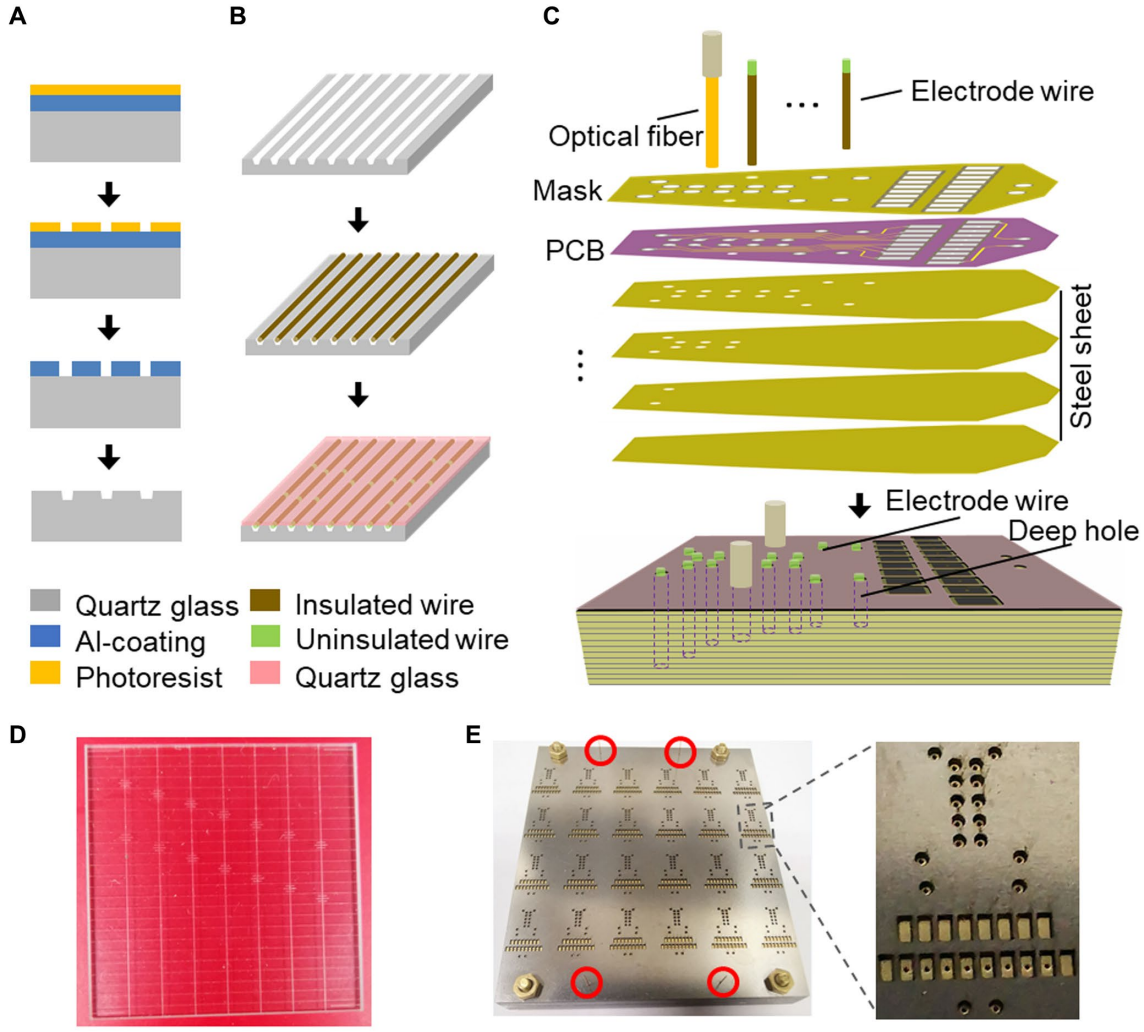
Fabrication process of the electrode mold. **(A)** Schematic illustration of the microwire mold using the dry etching. **(B)** Schematic illustration of the laser cutting and uninsulating in the microwire mold. **(C)** Schematic illustration of the process for electrode array fabrication on the laser drilling mold using the reflow soldering method. **(D)** A quartz glass mold with multiple microwire grooves. **(E)** Array of electrode molds. The red circles indicate the regions where the silicon tubes guide the alignment of stacked slices.

To facilitate the process of laser cutting and de-insulation of microwires, a microwire mold was designed. This mold was produced using two quartz plate parts: one with micro-grooves (Figures 1A,B,D) and one untreated quartz cover, obtained through micro-nanofabrication from Suzhou Rice-Semi Co., Ltd. in China. The production of the mold involved spin-coating a layer of photoresist onto an Al-coating quartz glass, followed by photolithography and bottom Al etching by phosphoric acid to obtain a micron-scale pattern. The exposed pattern on the quartz glass was then etched using CF4, and further mask stripped to create micro-grooves. The resulting mold contained multiple designed grooves, with each groove measuring 40 microns in width and 25 microns in depth.

For electrode microwire preparation (Figure 1B and Supplementary Figure S1), insulated Tungsten wire with a diameter of 33 microns (California Fine Wire) was used. Before being placed into the grooves, the wire was precut using a ceramic-coated scissor (#14958–09, Fine Science Tools). The microwire was then fixed in place with the untreated quartz cover. An optical fiber laser marking machine (Dezhou Qifu automation equipment Co., Ltd., China) was used to cut the microwires into the desired lengths within the grooves with high power output (90%) and to remove the insulated layer from the wire tips with low power output (60%). The laser scan speed was set at 500 mm/s, ensuring consistent and reliable microwire production.

#### Preparation of microwire bundles

To successfully record single-unit activities (SUAs) in targeted brain regions, it is crucial to construct microwire bundles that contain multiple microwires. To this end, we developed a matrix guide by fixing silica capillary tubes side by side (Supplementary Figure S2A). This design allowed for the efficient arrangement of microwire bundles, ensuring that each microwire was assigned a specific space within the bundle determined by the dimensions of the silica capillary tubes (Supplementary Figure S2B) and the length differences between each laser-cut microwire (Supplementary Figure S3C). The resulting microwire bundle could be arranged according to the specific experimental requirements (e.g., Supplementary Figure S2D), providing flexibility in our recording approach.

### Electrode positioning mold

In order to facilitate the assembly and welding of electrode wires and optical fibers, we have developed an electrode positioning mold (Figures 1C,E) to streamline the process. This mold is composed of cascaded steel sheets with parallel cylindrical cavities that have been laser-drilled according to the anterior–posterior and medial-lateral coordinates of the targeted brain regions, which were determined based on the horizontal coordinates of mouse DMN as specified in Table 1. The depth of each cavity corresponds to the thickness of the stacked laser-drilled steel sheets, as determined from the vertical coordinates of mouse DMN in Table 1. The electrode positioning mold enables us to assemble and position the electrode wires and optical fibers with precision according to the relative positions of the brain regions, which can then be fastened to a custom-printed circuit board (PCB) (Figure 1C and Supplementary Figure S3). To optimize efficiency, we utilize a batch-production mold (Figure 1E and Supplementary Figure S4), which streamlines the electrode production process and helps to ensure consistency and precision across experiments.

**Table 1:** Coordinates of the DMN nodes.

Regions	Paxino’s atlas			Steel stencil thickness/number
	AP	ML	DV	
Orb	2.2	±1	2.7	0.3/1; 0.2/1
PrL	1.9	±0.35	2.2	0.2/1
CG1	1.2	±0.35	1.9	0.1/1
CG2	0.5	±0.35	1.8	0.3/1
CG3	−0.2	±0.35	1.5	0.3/1; 0.1/1
RSC	−0.9	±0.35	1.1	0.3/1
S1TR	−1.5	±1.6	0.8	0.1/1
V2	−2.5	±1.5	0.7	0.1/1; 0.3/2
BF	0.1	±1.5	4.5	0.3/6

AP, anterior–posterior; ML, medial-lateral; DV, dorsal-ventral; Orb, orbital frontal cortex; PrL, prelimbic cortex; CG, cingulate cortex; RSC, retrosplenial cortex; S1, primary somatosensory cortex; V2, secondary visual cortex; BF, basal forebrain (location for optical fiber). The steel stencil number indicates the number of drilled steel stencils, which include all the AP/ML coordinates that the DV value is no less than the corresponding DV value. All units are in mm.

### Electrode assembling

To manually assemble the electrodes, the prepared microwires were threaded into the corresponding cylindrical cavity in the electrode positioning mold (Supplementary Figure S5A). After positioning all the microwires, solder paste was applied to the circuit board under the mask (Supplementary Figure S5B). The mask was then removed, and the connectors were placed on the pad while silver wires were inserted into the reference and ground channels. The microwires and connectors were reflow-welded (Supplementary Figures S5C,D). The mold and electrodes were then cleaned in an ultrasonic cleaner containing ethanol for 10 min to remove any residue from the scaling powder after welding. After air drying, optical fibers (Inper Co., Ltd., China) were threaded into the corresponding cylindrical cavity and bonded to the PCB using epoxy resin (9005, LEAFTOP, China). Once the UV curing was complete, solidified liquid photo solder resist (MECHANIC, China) was used to protect the optical fibers and insulate the soldering point (Supplementary Figure S5E).

In cases where microwire bundles were utilized instead of optical fibers for SUA recording, the microwire bundles were inserted into the cylindrical cavity designated for optical fibers and secured with epoxy resin. The uninsulated tips of the bundles were then folded and inserted into the corresponding welding hole before reflow-welding.

### Neuron labeling and electrode implantation surgery

**Figure 2:**
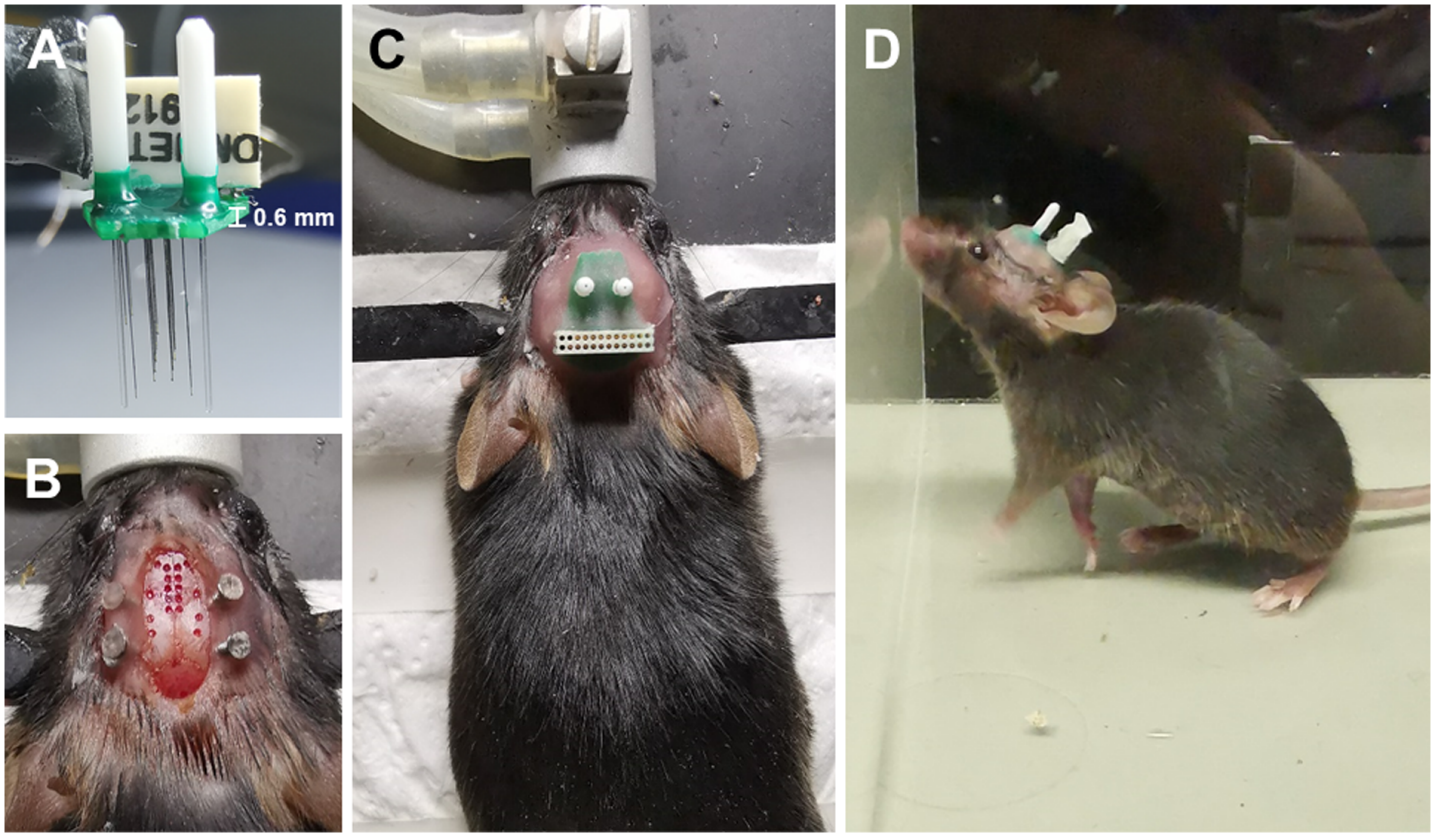
Electrode design is easy to implant. **(A)** An opto-electrode that has been assembled. **(B)** The implantation sites on the skull are exposed. **(C)** The electrode has already been fixed to the skull. **(D)** A free-moving mouse carrying the electrode.

In the surgical procedure, ChAT-Cre mice were anesthetized using 1%–2% isoflurane (RWD Life Science Inc., China) to maintain a respiratory rate of 40–50 breaths per minute. The animals were head-fixed using a stereotaxic apparatus with a water-circulating heating system (RWD Life Science Inc., China), and the temperature was set at 37°C. Following cranial adjustment, a cranial drill with a 400-micron diameter bit was attached to the arm holder of the stereotaxic apparatus. Bregma was used as the reference for zero setting, and the drilling process was initiated gradually, following the coordinates of the targeted brain regions. The dura was removed, and four stainless screws were tapped into the skull and reinforced with dental cement (Figure 2B). In addition, two screws were placed onto the cerebellum as reference and ground. The electrode was mounted onto an electrode holder, fixed to the stereotaxic apparatus arm, and the microwires/fibers were aligned with their designated holes. The stereotaxic arms were gradually lowered until the longest microwire/fiber contacted the brain surface at the center of the hole, and the Z arm was then set at zero before further lowering the electrode to the desired depth. Gel foam was applied to all drilled holes. After the implantation, the opto-electrode was fixed to the skull using dental cement. The mice were caged individually to recover before recordings.

To genetically label the cholinergic neurons in the basal forebrain (BF), we injected a titer (0.1 μL, 3 × 10^12^ genomic particles/ml) of the rAAV2/9-hSyn-DIO-ChR2-GFP virus into the brain region using a pump (UMP3-MICRO4, World Precision Instruments; 50 nL/min) with a flow rate of 50 nL/min, followed by a 15 min maintain period.

### Electrophysiology recording

We conducted recordings of both electrophysiological and behavioral data using a 32-channel electrophysiology system from Plexon (United States). The signal was sampled at 1,000 Hz and 40,000 Hz, with a 0.05 Hz high-pass filter applied for the LFPs and individual spikes recording. To monitor head movement, we attached a 3-axis acceleration sensor from FDISYSTEMS (China) to the pre-amplifier. We also synchronized the video (from Plexon, United States) and acceleration at 30 frames and 20 Hz, respectively. All experiments were conducted in a noise-attenuated room with an environmental background noise of 32.2 ± 3.0 dB (mean ± SD).

### Steady state visually evoked potential (SSVEP)

To evoke neural activity, we delivered an 8 Hz flash stimulation above the homecage while concurrently recording electrophysiological data for further analysis.

#### Optogenetics

In order to activate cholinergic neurons located in the basal forebrain (BF), we employed a 473-nm sinusoidal wave laser (DPSS laser, Inper Co., Ltd) operating at 8 Hz and with a power output of 3 mW for light stimulation. Concurrently, we recorded electrophysiological data to enable further analysis.

#### Histology

To validate virus expression and the precise location of the implanted electrode/optical fiber, all animals were perfused after the experiment. Mice were deeply anesthetized and subjected to intracardial perfusion with saline (0.9% w/v NaCl) followed by 4% Paraformaldehyde (PFA) solution. The brains were extracted and post-fixed in 4% PFA. Subsequently, 30 μm-thick coronal sections were prepared using a Leica Microsystems (Wetzlar, Germany) microtome after gradient dehydration with sucrose (15% and 30%). For immunofluorescence staining, free-floating brain sections were first blocked with 3% normal goat serum at room temperature for 1 h. Next, they were incubated with anti-ChAT (1:200, #AB144P Millipore) and conjugate-adsorbed Alexa Fluor 546 donkey anti-goat (1:500, #A-21432 Invitrogen). After immunofluorescence staining, the sections were additionally stained with DAPI, rinsed, dried, and cover-slipped with a fluorescence mounting medium. Finally, the sections were examined under a confocal laser-scanning microscope (Zeiss LSM800, Germany).

### Data processing

In this study, data processing was carried out using internal Matlab scripts (release 2021b) and EEGLAB (Delorme and Makeig, 2004). Power spectral density estimation and time-frequency analysis were performed using the PWELCH and CWT functions, respectively. To prevent memory overflows, the data were segmented into periods during time-frequency estimation. Spike sorting was conducted using Offline Sorter, a commercial software from Plexon (United States). The firing rate, valley-to-peak time, and burst index of the recorded spike units were calculated and analyzed. The firing rate was determined by dividing the total firing number by the total duration of the behavioral state and was used for further analysis.

### Statistical analysis

In this study, we conducted significance testing using paired two-tailed Student’s *t*-test and considered *p*-values less than 0.05 as statistically significant. The statistical analyses were performed using GraphPad Prism software (GraphPad Software, Inc., San Diego, CA).

## Results

### Design concept and electrode structure

The implantation and fixation of microwires/optical fibers for multiple mouse brain regions presented significant challenges. To overcome these challenges, we developed a potential solution to fabricate an opto-electrode that would allow for the fixation of all microwires/optical fibers for multiple brain regions into a single module. We designed and developed a mold consisting of a series of stacked slices (Figure 1) that would enable the presetting of microwires/optical fibers in the 3D space of the target brain regions and connect them to a connector. The mold comprises 2D horizontal (X and Y) planes that form the 3D shape along the Z direction, creating cylindrical cavities at each designed location for the placement of the microwires/optical fibers (Figure 1C). We utilized PCBs to fix and connect the microwires/optical fibers (Figures 1C,E), in which the de-insulated parts of the microwires were soldered to the PCB pads (Figure 1B). The micro-nanofabrication quartz glass was utilized to fix the microwires (Figures 1A,D) due to its excellent high-temperature resistance and infrared transmittance. After assembling the opto-electrodes, UV-curable resin was used to insulate and protect the PCB. The resulting opto-electrode was a lightweight (0.28 g) and small space-size (10 mm length, 8 mm width, and 7 mm height) multi-channel opto-electrode that allowed for the synchronized recording and stimulation of distributed brain regions (Figure 2A). This electrode is easy to implant and carry by mice without difficulty (Figures 2B–D).

### Electrode placement in brain regions

**Figure 3:**
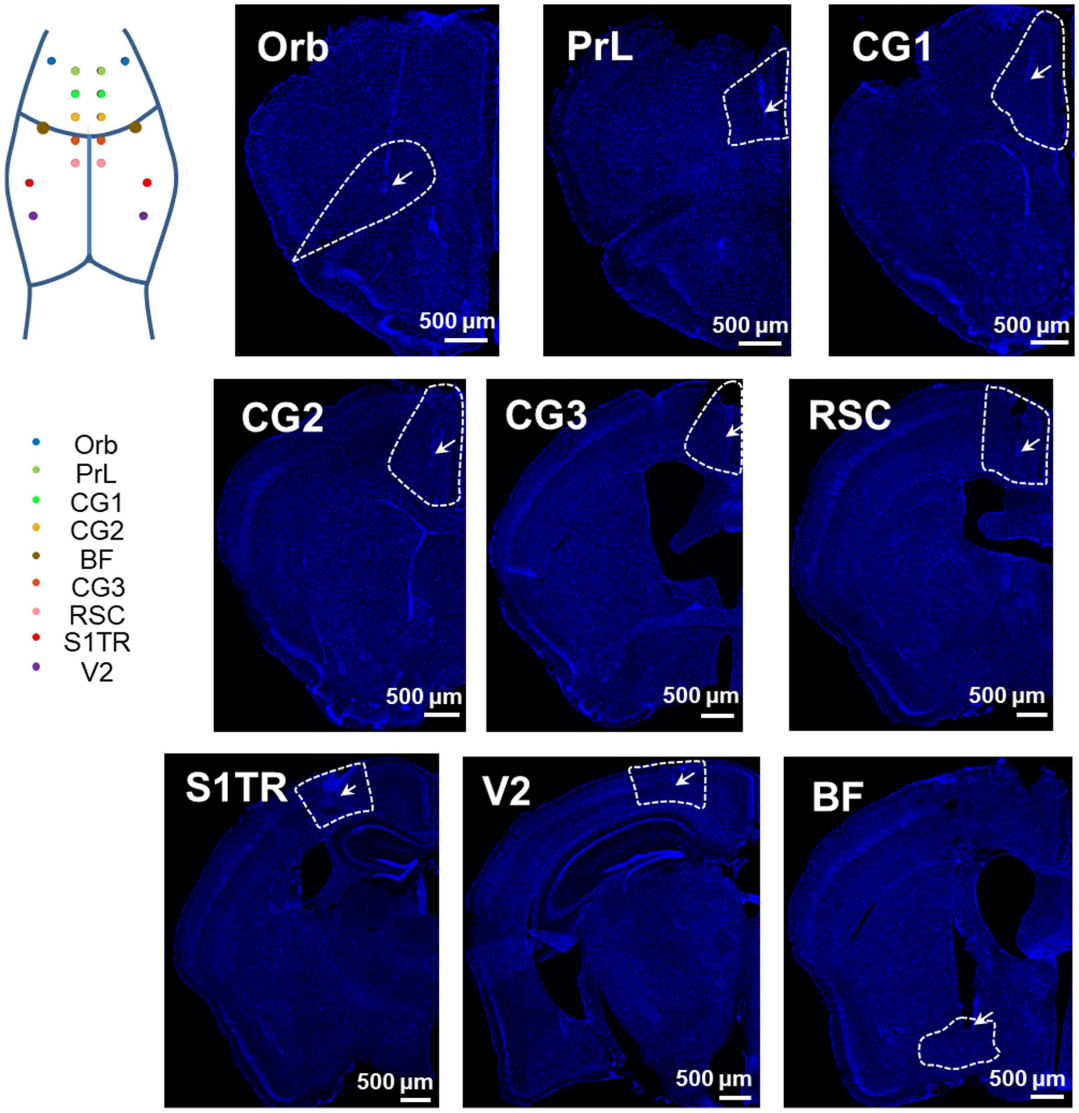
Positioning accuracy of electrodes in multiple brain regions. Representative images illustrate the electrode locations in all implanted regions (stained with DAPI). The target DMN regions are depicted as wireframes, the tips of microwires or fibers are labeled with arrows, and the white bars indicate a length of 500 microns. The map in the top left corner shows the implantation sites in an illustrative manner.

To assess the accuracy of our opto-electrode, we implanted the opto-electrodes in 16 DMN and 2 basal forebrain (BF) regions of mice. Subsequent histological analysis confirmed that the microwires were implanted in the central of the targets, and the optical fibers were implanted in the upper-edge of the targets, as illustrated in Figure 3. These results demonstrate the accuracy and reliability of our opto-electrode and implantation technique.

### High-quality electrophysiology data recorded

**Figure 4:**
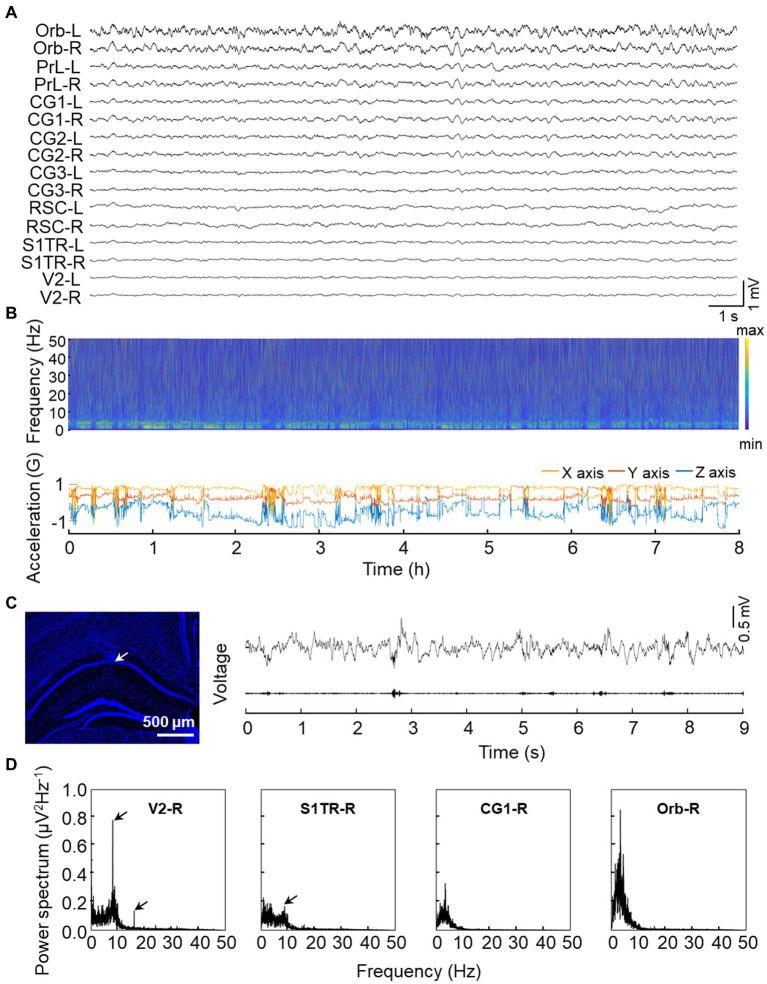
Validation experiments of signal quality from implanted electrodes. **(A)** Representative raw signal trace of an 8-s window. **(B)** Representative long-term electrical activity. The upper panel displays the LFP spectra of CG1-L while the bottom panel shows 3-axis acceleration recorded by the on-head accelerometer. Multiple rest/sleep cycles are visible from the synchronous recording. **(C)** Representative high-frequency ripple activity recorded from CA1. The left panel shows the electrode location in the CA1 region with the tip of the microwire indicated by the arrow and a white bar indicated with 500 microns. The upper trace of the right panel displays the raw LFP activity recorded from CA1 while the bottom trace of the right panel shows the 100–250 Hz bandpass filtered signal. **(D)** Representative low frequency (8 Hz) SSVEP recorded from DMN regions. Power spectral density of a 3-min time window is shown with arrows indicating the 8 Hz or its harmonic response.

To evaluate the functional characteristics of our opto-electrode, we implanted it into three mice for an extended period of time. After 24 h of implantation, we observed steady electrical activity, and all electrode channels functioned correctly for at least 4 weeks without any observed movement difficulties in the animals. We conducted long-term recordings lasting over 8 h to assess the stability of the electrode. We demonstrated that the recording is relatively unaffected by movement noise, as shown in the recording sample of a freely moving mouse depicted in Figure 4A, where the stable spectra fluctuations match the head movement of the mouse during the lengthy recording session (Figure 4B). Additionally, we implanted the electrode into CA1, a region known to generate high-frequency activity (ripple oscillations) during cognitive activities (Buzsaki, 2015), and observed prominent ripple activities as shown in Figure 4C. To evaluate low-frequency activity recording, we used SSVEP (Xu et al., 2013; Yan et al., 2017) and observed a strong power response in response to low flash stimulus frequency (8 Hz) in the visual-related pathway (Figure 4D).

**Figure 5:**
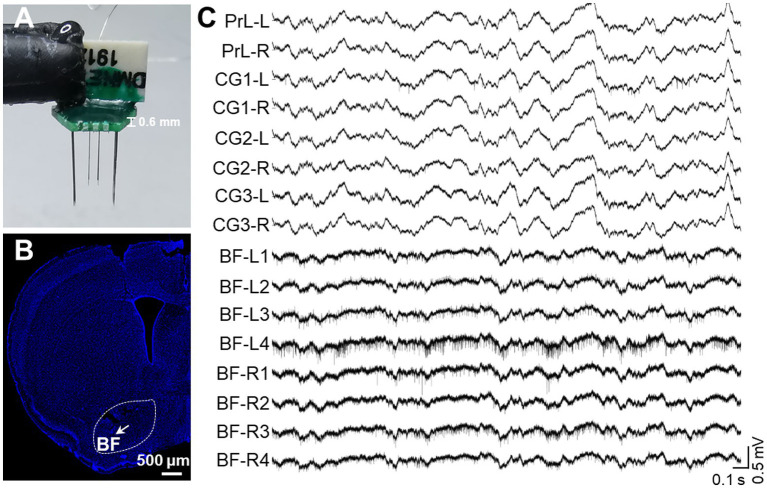
*In vivo* recording of multiple single-unite activities in key brain regions. **(A)** An assembled electrode for key DMN regions and BF. A total of 16 channels includes 8 channels for bilateral BF (4 channels on one side) and 8 channels for bilateral PrL, CG1, CG2, and CG3. **(B)** A representative image of the electrode array location in BF. The arrow indicates the position of the electrode tip and the white bar indicates 500 microns. **(C)** Representative raw signal trace of a 3-s window for key DMN regions and BF.

**Figure 6:**
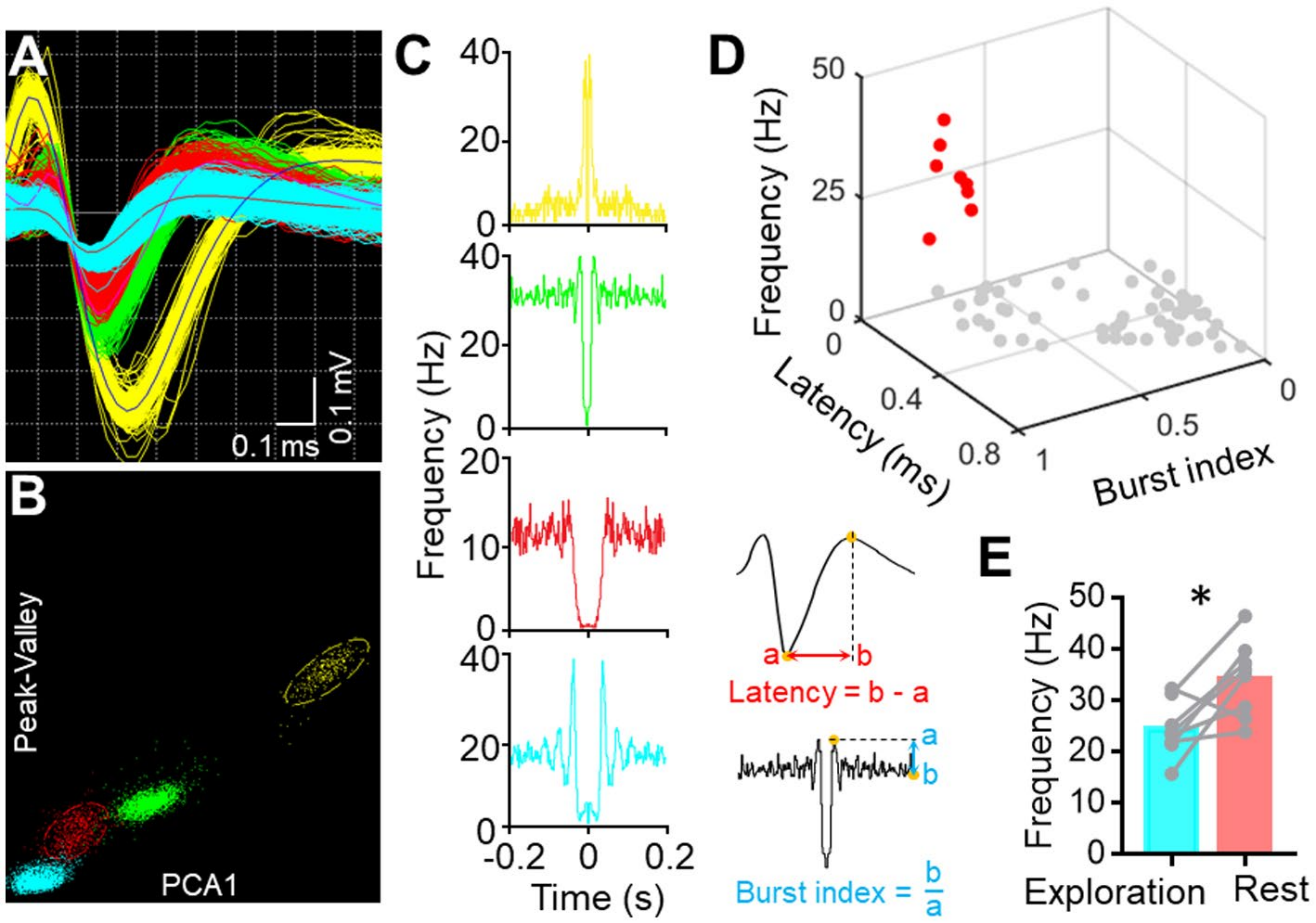
Single-unit sorting. **(A)** Representative image displays the waveform of four sorted single units from one BF channel. **(B)** Representative image shows the clustering of four sorted single units based on their Peak-Valley and PCA1 characteristics. **(C)** Auto-correlogram of each single-unite activity. **(D)** Scatter diagram illustrates the features of each sorted unit (*n* = 67, pooled from three mice) from BF. Red dots represent the putative PV+ neurons. **(E)** Graph shows the firing frequency of PV+ neurons in BF during free exploration and rest (*p < 0.05, *n* = 8 neurons from three mice, paired *t*-tests).

Single-Unit Activity (SUA) carries rich information associated with brain functionality (Remondes and Wilson, 2013; Tingley and Buzsaki, 2018). Therefore, we implanted another version of the electrode with microwire bundles to record the discharging of single neurons in BF and key DMN regions. Despite the large variance in channel discharges due to state changes, most of the recording channels captured single-unit activity, as indicated in Figure 5. BF activity is known to influence animal behavior, particularly parvalbumin-positive (PV^+^) neurons, with high activity promoting rest behavior in rodents (Lozano-Montes et al., 2020). Therefore, we isolated PV^+^ neurons from the recorded neurons based on their electrophysiological characteristics (Figures 6A–D). The firing rate of putative PV^+^ neurons in BF increased during resting behavior compared to exploration behavior (Figure 6E), consistent with previous studies (Lozano-Montes et al., 2020). Most single cell activities can be continuously detected for a minimum duration of 5 days, and the rest of the electrophysiological activities can be stably detected for at least 4 weeks. These data demonstrate that our electrode is capable of high-quality electrophysiological recording.

### Electrophysiological responses to blue light activation

**Figure 7:**
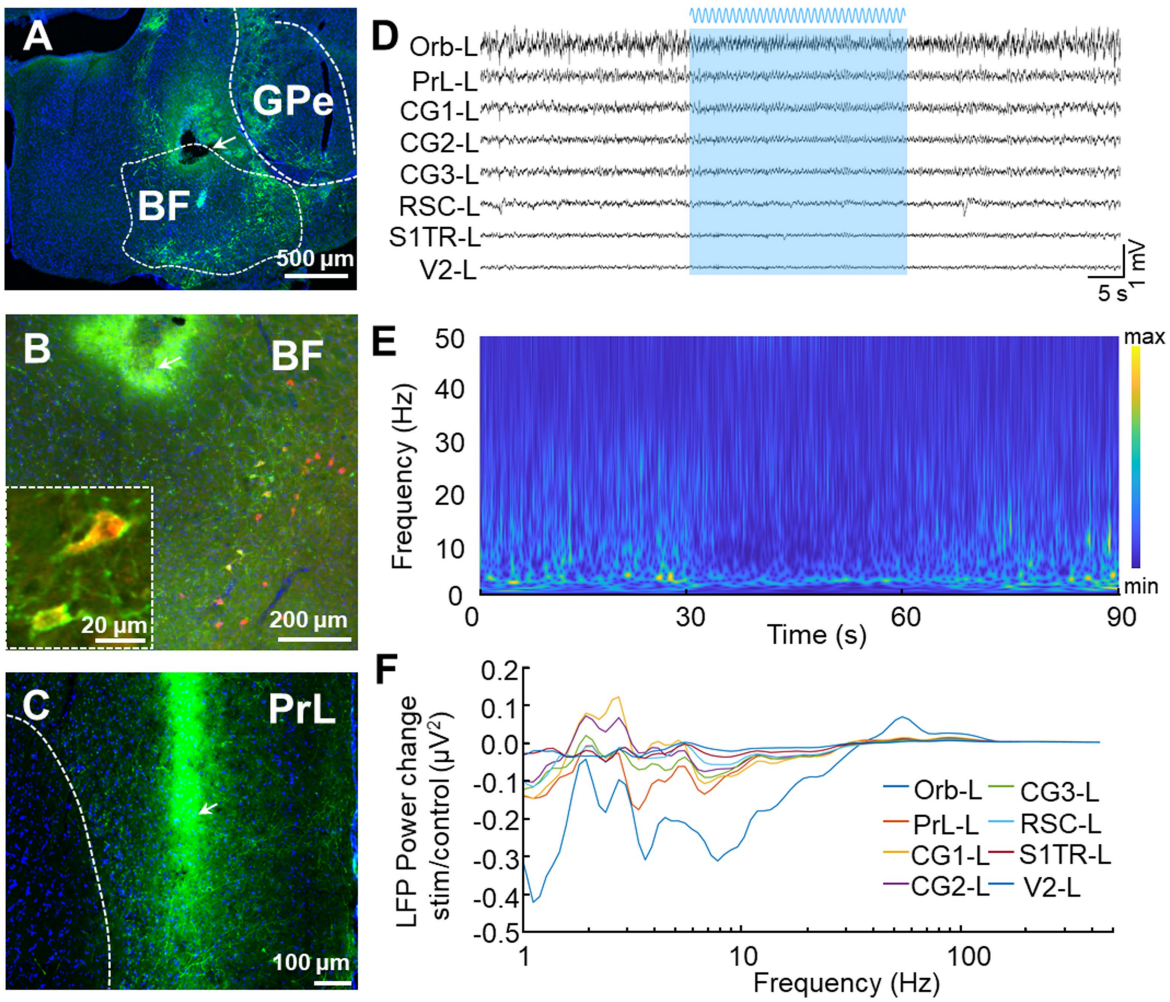
*In vivo* recording of LFP signals with optogenetics stimulation. **(A)** Representative image displays the expression of ChR2-GFP after the virus rAAV2/9-hSyn-DIO-ChR2-GFP was injected into the BF of ChAT-Cre mice. The tip of the fiber is indicated by the arrow. The white bar represents 500 microns. **(B)** Low and high magnifications of the images show that all the ChR2-GFP^+^ cells are ChAT^+^ cells (red). The white bars represent 20 (left) and 200 (right) microns, respectively. **(C)** Representative image displays the distribution of GFP terminals and electrode location in PrL. The arrow indicates the position of the electrode tip. The white bar represents 100 microns. **(D–F)** Stimulation of BF elicits a selective effect on DMN frequencies. **(D)** An example LFP from left DMN regions. The blue area represents BF sinusoidal blue light stimulation. **(E)** Power spectrograms of PrL-L. A decrease in activities around theta frequency band was observed during light stimulation. **(F)** LFP power spectrograms in left DMN regions were modulated by sinusoidal blue light stimulation.

We conducted further analyses on the blue light activating LFP responses (Figure 7) by injecting the Cre recombination-dependent rAAV2/9-DIO-ChR2-GFP virus into the BF of ChAT-Cre mice before placing optical fibers above these brain regions (Figure 7A). This virus was used to specifically express channelrhodopsin-2-H134R (ChR2, a modified version of a light-gated ion channel) in cholinergic neurons of the BF. We used the ChAT-Cre mouse model because cholinergic neurons in the BF innervate most of the DMN regions (Do et al., 2016). We confirmed the specific labeling of ChAT neurons (ChAT^ChR2-GFP^) through immunofluorescence staining and found that nearly all of the GFP neurons (94.9 ± 1.3%, n = 3) were ChAT positive (Figure 7B). To assess the modulation of DMN activity resulting from the activation of cholinergic neurons in the BF, we applied blue laser light onto ChAT^ChR2-GFP^ neurons in mice BF to examined the resulting LFP responses in DMN regions. We found a decrease in theta-band oscillation in almost all DMN regions during sinusoidal stimulation in BF (Figures 7C–F), validating the modulation of DMN activity by cholinergic neurons in the BF.

## Discussion

We developed an opto-electrode that can be easily mass-produced and enables simultaneous electrophysiological recording and optical stimulation in 18 widely separated and interconnected brain regions of freely moving mice. Our design employs laser-cut microwires and laser-drilled molds, which enhances assembly efficiency and accuracy. The ultra-lightweight and compact size of the opto-electrode ensures its easy implantation and long-term stability during stimulation/recording. We implanted microwires/optical fibers into 16 DMN and 2 BF regions of the mouse brain and demonstrated successful simultaneous recording/stimulation.

### Comparison with other multisite penetrating electrodes

Our presented opto-electrode possesses several advantages, including its ultra-lightweight and compact size, as well as its ability to record and stimulate a larger number of brain regions while maintaining comparable LPF/SUA recording performance to previous electrodes (Headley et al., 2015; Billard et al., 2018; Ma et al., 2019; Sheng et al., 2021). The opto-electrode weighs only 0.28 g, which enables it to cover up to 18 brain regions while reducing the burden on animals to tolerate and behave freely without excessive physical exertion, even when a cable suspension system is not utilized to balance the weight (Battaglia et al., 2009). Moreover, the smaller size of our opto-electrode design reduces limitations on animal behavior. Unlike other multi-drive-based movable electrodes (Headley et al., 2015; Billard et al., 2018; Ma et al., 2019; Sheng et al., 2021), our opto-electrode does not require additional space to accommodate and fix the drives, thus reducing the horizontal sloshing caused by a higher electrode center of gravity and lower torque imposed during the animal’s motion. Additionally, the opto-electrode’s smaller size and weight allow for the utilization of a larger capacity battery in wireless stimulation/acquisition systems (Szuts et al., 2011; Gagnon-Turcotte et al., 2017; Shin et al., 2022), which provides longer recordings without increasing the overall weight.

### Limitations

Although the materials used in current electrodes are cheap and readily available, the high Young’s modulus of the metal microwire and quartz optical fiber could lead to persistent tissue damage and immunoreactive glial responses in the brain (Cao et al., 2021; Zhou et al., 2022). These factors could significantly impact the chronic stability of recording, particularly the single-unit activity. However, the current opto-electrode design incorporates flexible materials, which should help to reduce damage and increase the stability of long-term recordings. In addition, while the opto-electrode described in this study utilizes a laser processing mold that permits mass assembly while retaining high assembling accuracy, achieving a spatial precision of approximately 20 μm (Ma et al., 2019), there still exists a possibility of mispositioning of the microwires during implantation. It is particularly prominent when only one microwire is designed for one brain region, as it can drift to the side. However, this effect can be significantly reduced by increasing the number of microwires or by enhancing the strength of a single microwire.

## Conclusion

In conclusion, our study offers a novel opto-electrode solution for recording/ stimulating up to 18 brain regions in freely moving mice. The compact size and lightweight design of the electrode enable efficient experiment handling and reduced burden on the animal. Furthermore, the flexibility of the electrode design allows for the customization and targeting of specific brain regions, enabling the investigation of neural circuits and networks.

## Data availability statement

The raw data supporting the conclusions of this article will be made available by the authors, without undue reservation.

## Ethics statement

The animal study was reviewed and approved by Animal Care and Use Committee of the animal core facility at Huazhong University of Science and Technology.

## Author contributions

HD and YL conceived and designed the studies and wrote the paper. QZ and WJ carried out the experiments including electrode fabrication, electrophysiology recording, and data analysis. SW, MZ, JJ, XL, DY, LC, BF, JW, and FX performed the experiments including genotyping, PCR immunofluorescence, and cell counting. All authors contributed to the article and approved the submitted version.

## Funding

This work was supported by the National Natural Science Foundation of China (Grants: 31900734 to HD; 81901366 to WJ; 31721002 to YL; 81920208014 to YL; and 31930051 to YL).

## Conflict of interest

The authors declare that the research was conducted in the absence of any commercial or financial relationships that could be construed as a potential conflict of interest.

## Publisher’s note

All claims expressed in this article are solely those of the authors and do not necessarily represent those of their affiliated organizations, or those of the publisher, the editors and the reviewers. Any product that may be evaluated in this article, or claim that may be made by its manufacturer, is not guaranteed or endorsed by the publisher.
