# Supplementary material for: Development of a synchronous recording and photo-stimulating electrode in multiple brain neurons
Source: Front Neurosci. 2023 Jun 13;17:1195095. doi: 10.3389/fnins.2023.1195095 (PMC10293621; doi:10.3389/fnins.2023.1195095)
Supplement: Supplementary file 1 [file Data_Sheet_1.PDF]

Fig. S1 Laser cutting microwire.

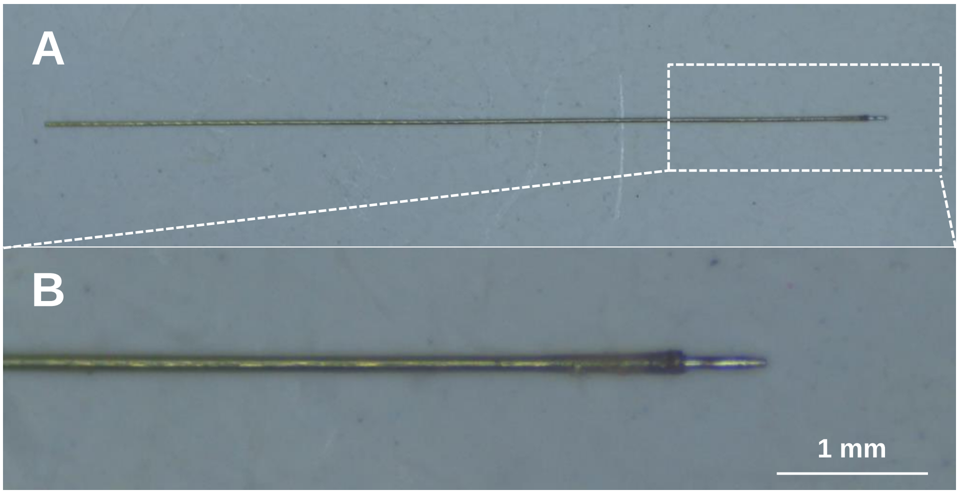

Fig. S1 Laser cutting microwire. Related to Figure 1.

(A and B) Low (A) and high (B) magnifications of the image show the laser cutting and de-insulation of one microwire. The white bar indicates 1 millimeter.

Fig. S2 Preparation of microwire bundles.

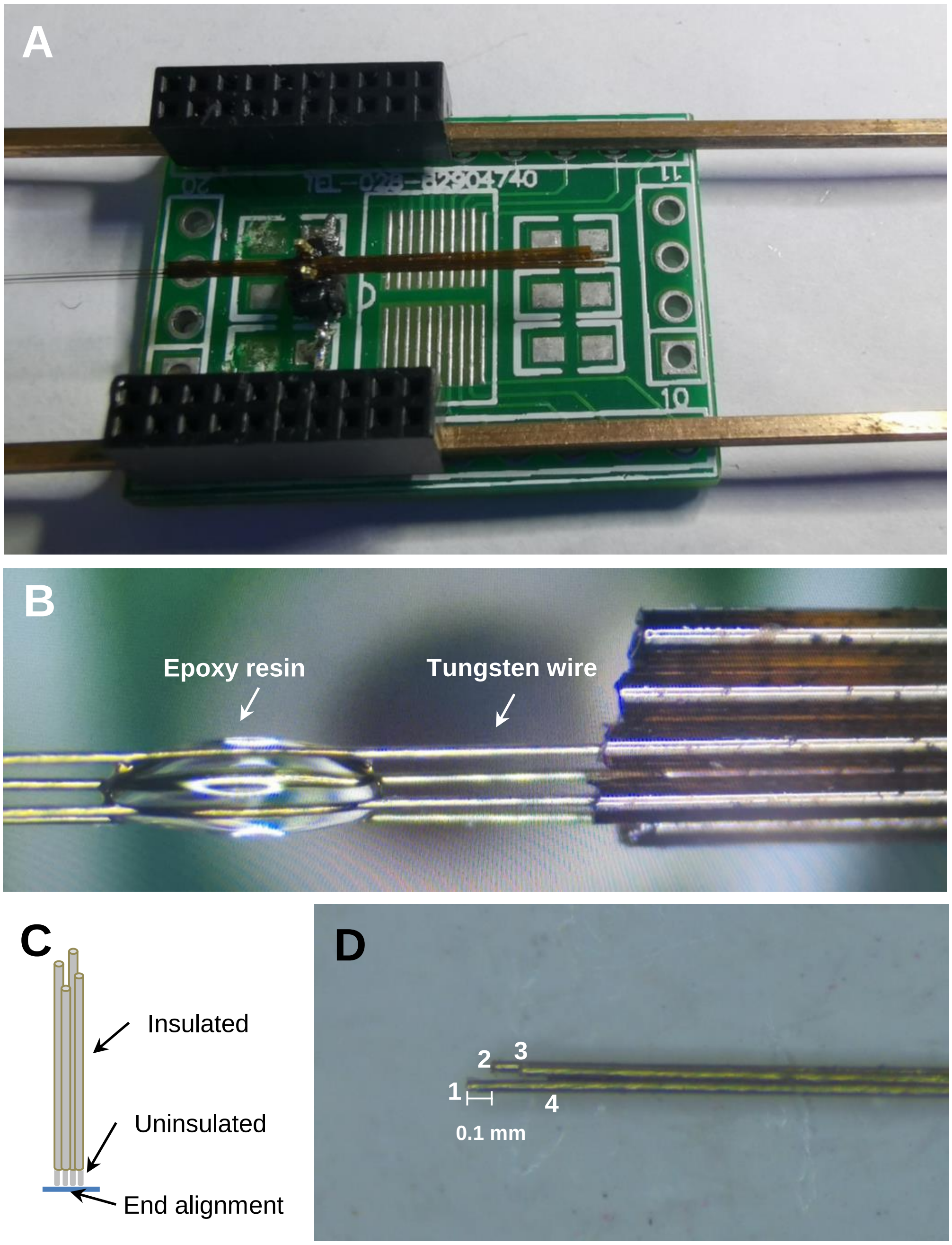

Fig. S2 Preparation of microwire bundles. Related to Figure 1.

(A) A 2*4 silica capillary tube matrix for microwire guide.

(B) A 2*2 tungsten wire bundle fixed with epoxy resin.

(C) Schematic illustration of the tips space ensures end alignment .

(D) The tip configuration of one 2*2 microwire bundle.

Fig. S3 PCB layout.

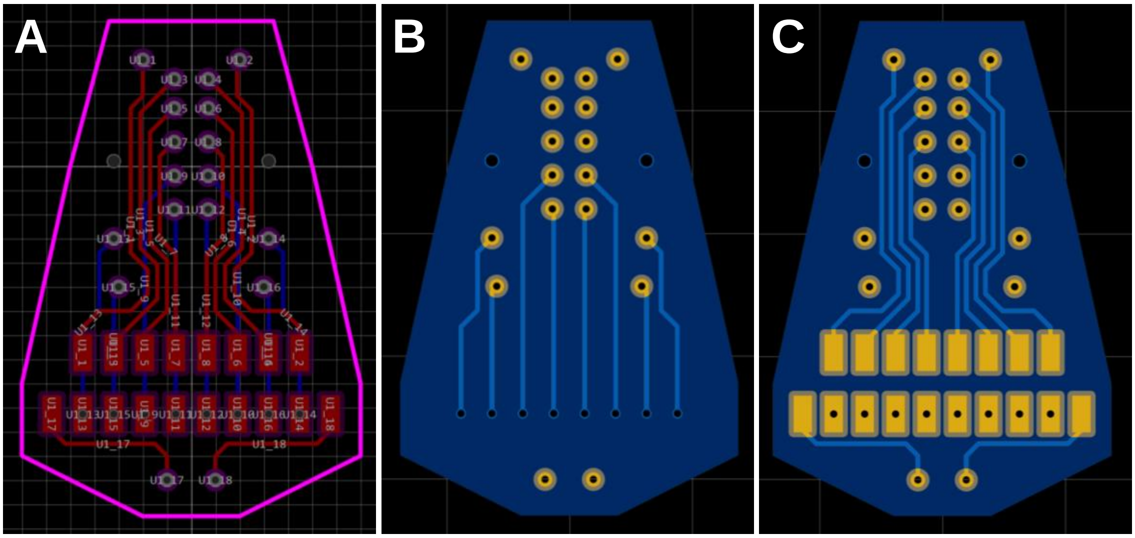

Fig. S3 PCB layout. Related to Figure 1.

(A) All layers of PCB.

(B) Preview of PCB bottom layer.

(C) Preview of PCB top layer.

Fig. S4 Die component.

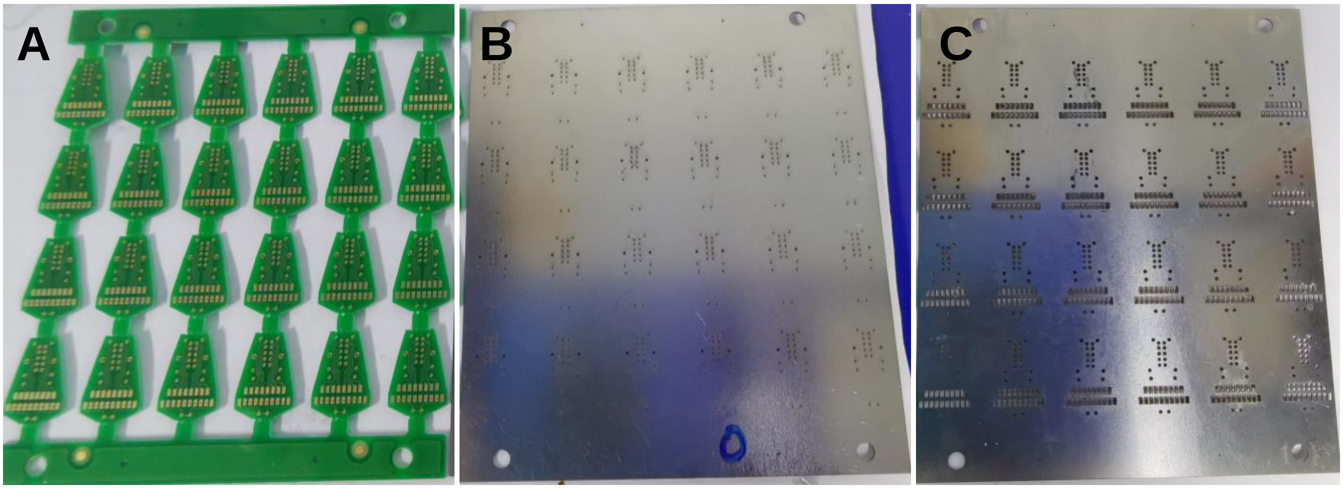

Fig. S4 Die component. Related to Figure 1.

(A) Circuit board array.

(B) Drilled steel sheet.

(C) Mask.

Fig. S5 Critical process of electrode assembling.

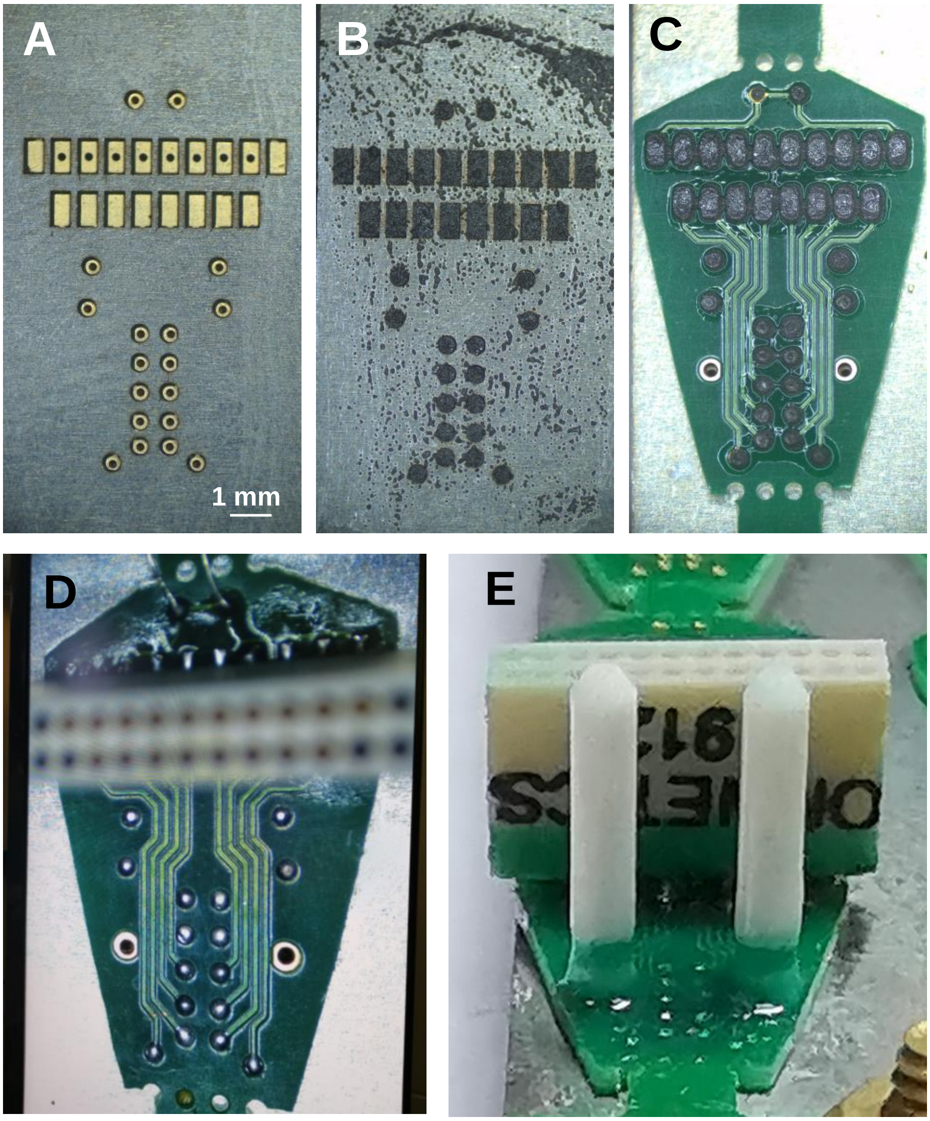

Fig. S5 Critical process of electrode assembling. Related to Figure 1.

(A) Penetration microwire. The white bar indicates 1 millimeter.

(B) Coating solder paste.

(C) Remove mask.

(D) Reflow welding.

(E) Penetration of optical fiber and reinforcement.
